# Correlations Between Vestibular Function and Imaging of the Semicircular Canals in DFNA9 Patients

**DOI:** 10.3389/fneur.2019.01341

**Published:** 2020-01-10

**Authors:** Berina Ihtijarevic, Sebastien Janssens de Varebeke, Griet Mertens, Sven Dekeyzer, Paul Van de Heyning, Vincent Van Rompaey

**Affiliations:** ^1^Department of Otorhinolaryngology and Head and Neck Surgery, Antwerp University Hospital, Antwerp, Belgium; ^2^Department of Otorhinolaryngology and Head and Neck Surgery, Jessa Hospital, Hasselt, Belgium; ^3^Department of Translational Neurosciences, Faculty of Medicine and Health Sciences, University of Antwerp, Antwerp, Belgium; ^4^Department of Radiology, Antwerp University Hospital, Antwerp, Belgium

**Keywords:** DFNA9, electronystagmography, semicircular canal, magnetic resonance imaging, computed tomography

## Abstract

**Background and Purpose:** Radiologic abnormalities on computed tomography (CT), including narrowing or sclerosis of the semicircular canals (SCCs), and T2-weighted magnetic resonance imaging (MRI), including signal loss in the SCC, have been reported as potential biomarkers in patients with P51S mutations in the *COCH* gene (i.e., DFNA9). The aim of our study was to correlate caloric responses through electronystagmography (ENG) data with imaging results in DFNA9 patients.

**Materials and Methods:** A retrospective study was performed in 45 patients; therefore, 90 ears with P51S mutations in the *COCH* gene were tested. Caloric responses and CT and MRI data were analyzed from June 2003 until May 2014. More than half of patients (54%) were candidates for cochlear implantation.

**Results:** In our population, 91% of tested ears had sclerotic lesions and/or narrowing in one or more SCCs on CT scan. All tested ears had narrowing or signal loss in at least one SCC on T2-weighted MRI. The lateral SCC was affected in 87% on CT scan and 92% on MRI. However, in 83% of tested ears, all three SCCs were affected on MRI. Furthermore, in 77% of tested ears, caloric responses were reduced bilaterally, while 11.5% showed unilateral hypofunction and the other 11.5% had normal caloric responses. CT abnormalities correlated with hypofunction of caloric responses. This statistically significant difference was present if abnormalities were observed in at least one of the SCCs as well as in ipsilateral lateral SCC function loss. MRI abnormalities in at least one of the SCCs correlated with ENG hypofunction, but there was no direct correlation between lateral SCC abnormalities on MRI and caloric responses of the investigated lateral canal.

**Conclusion:** Our retrospective analysis confirms the presence of CT and MRI abnormalities in DFNA9 patients with the P51S mutation in the *COCH* gene. A correlation between these radiologic features and vestibular function (tested by means of caloric response) was found in this population.

## Introduction

DFNA9 is an autosomal dominant hereditary disorder characterized by progressive vestibular and cochlear deterioration. Patients typically become symptomatic in the third to fourth decade of life and usually present with oscillopsia and unsteadiness in darkness, due to bilateral vestibulopathy (1, 2). In a later stage, they develop severe-to-profound sensorineural hearing loss (3). DFNA9 is a non-syndromic form of hearing loss caused by a mutation in the coagulation factor C homology (*COCH*) gene (4). Many different mutations have been identified in the *COCH* gene worldwide, where P51S is a frequent mutation in Belgium and the Netherlands (4, 5). Histopathological studies show accumulation of eosinophilic glycosaminoglycan deposits with misfolded cochlin, which causes atrophy of fibrocytes, especially in the spiral ligament and limbus of the cochlea as well as the crista ampullaris of the semicircular canal (SCC) and the maculae of the vestibular system. This loss of fibrocytes leading to accumulation of acellular substance that probably consists of misfolded *COCH* protein may be the cause of cytotoxicity (6–8). Radiologic abnormalities on computed tomography (CT), including narrowing or sclerosis of the SCC, and T2-weighted magnetic resonance imaging (MRI), including signal loss in the SCC, have already been reported as a biomarker in carriers of the P51S mutations in the *COCH* gene (9). The authors have shown a correlation of these lesions with advanced stages of sensorineural hearing loss, suggesting these lesions to be secondary to an advanced inflammatory process.

The aim of our study was to correlate vestibular function, through caloric responses during electronystagmography (ENG) with CT and MRI abnormalities in the SCCs in DFNA9 patients.

## Materials and Methods

### Ethics

The study was designed and conducted according to the Declaration of Helsinki (1996). The study was approved by the local ethics committee of the Antwerp University Hospital/University of Antwerp (protocol number 16/42/426).

### Study Design

A retrospective study was performed in 45 patients with P51S mutations in the *COCH* gene, where 90 ears in total were investigated. ENG caloric responses and CT and MRI data were analyzed from June 2003 until May 2014. Both CT and MRI data were blinded to the investigators. A consultant neuroradiologist assessed all the CT and MRI blinded to the electronystagmography data.

### Setting

Single tertiary referral otology department.

### Audiometry

Hearing loss was defined by pure tone audiometry showing air-conducted hearing thresholds averaged for 500, 1,000, and 2,000 Hz higher than 16 dB HL (10). A subgroup of patients underwent evaluation for cochlear implantation candidacy. The criteria for reimbursement of cochlear implantation in Belgium are as follows: pure-tone average of 500, 1,000, and 2,000 Hz in unaided liminal audiometry exceeding 85 dB and speech discrimination with hearing aid <30%.

### Vestibular Function Testing

Bilateral caloric irrigation was used to evaluate lateral SCC function. The methodology and normative values were reported earlier by Van der Stappen et al. (11) To summarize, subjects were seated in complete darkness, in a supine position with a head incline of 30°. Bithermal caloric irrigations (30°/44°) were performed in a 30 s time span, and nystagmus was recorded using ENG (Nystagliner Toennies, Germany). Caloric responses were categorized as follows:

- Caloric areflexia: sum of bithermal, 30° and 44°, max. peak slow phase velocity (SPV) < 6°/s per ear, in accordance with the Barany Society criteria for bilateral vestibulopathy (12).
- Caloric hypofunction: sum of bithermal, 30° and 44°, max. peak SPV < 10°/s and >6°/s.
- Normal caloric response: sum of bithermal max. peak SPV > 10°/s.

### Computed Tomography

**Figure 1:**
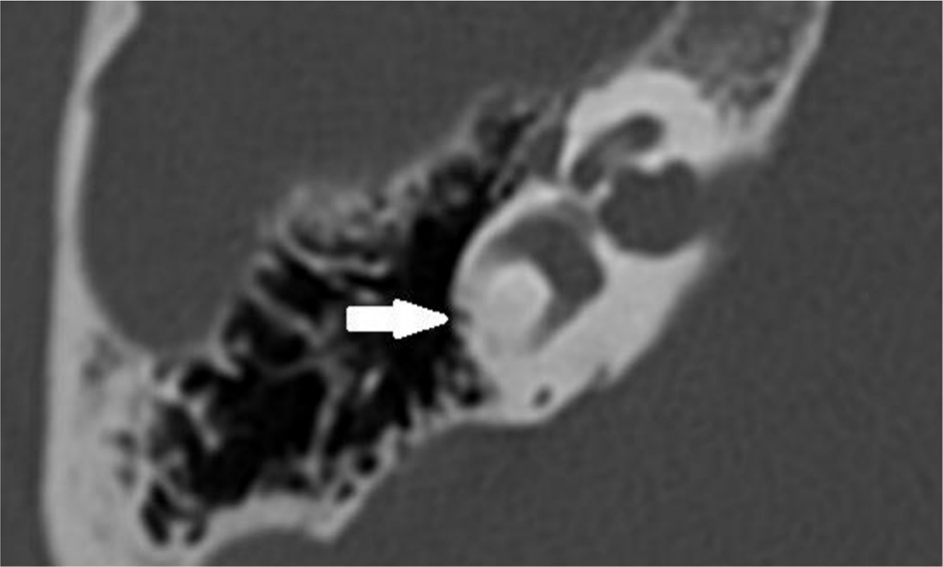
Axial temporal bone CT scan of the right labyrinth demonstrating narrowing and ossification of the lateral semicircular canal (arrow).

Multi-slice helical CT imaging of the temporal bone was performed on a 64-section CT scanner (LightSpeed VCT, GE Healthcare) with a 0.625 mm helical thickness. Tube voltage was 140 kV with a charge of 330 mA. A pitch of 0.531 mm per rotation was used with a rotation time of 1 s and an interval of 0.321 mm. Total acquisition time was 5.75 s. A field of view of 250 mm was used. Multiplanar reformation was performed with axial images reconstructed in the plane of the lateral SCC and coronal images reconstructed perpendicular to this plane. Additional reconstruction parallel to the superior SCC (Pöschl's plane) was performed. These reformations had a slice thickness of 0.2 mm with a field of view of 96 mm. Narrowing or sclerosis at the level of the SCC on CT imaging was defined when there was <50% of normal SCC diameter compared to the normal side or in consensus in case of bilateral pathology (Figure 1).

### Magnetic Resonance Imaging

**Figure 2:**
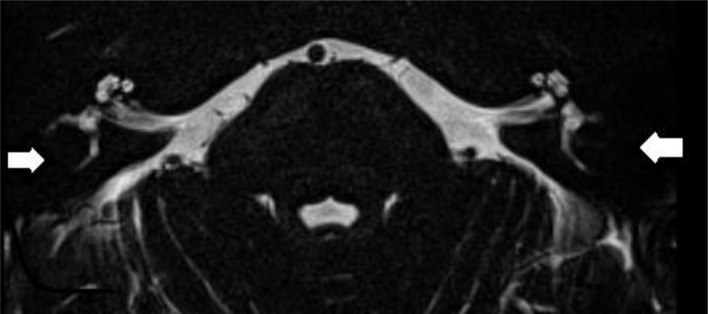
Axial T2-weighted turbo spin echo MRI of the left and right labyrinth demonstrating signal loss in the lateral semicircular canal (arrow) corresponding to the axial CT slice in Figure 1.

MRI scans were performed on a 3-T system (Siemens Magnetom TIM Trio or Siemens Magnetom Skyra, Erlangen, Germany). The patients were positioned with their head in a 32-channel circularly polarized head coil. For each patient, an MRI scan of the brain and skull base was accomplished using the following sequences: axial T2-weighted turbo spin echo (T2 TSE) images and axial Fluid Attenuated Inversion Recovery (FLAIR) through the entire brain, followed by a 3D-turbo spin echo: “Sampling Perfection with Application optimized Contrasts using different flip angle Evolution” (SPACE) with TR/TE = 1,000/129 ms, 0.5 mm isometric voxels, a field of view of 200 mm, and a 384 × 384 matrix through the skull base. Also, sequences after gadolinium were included. The high spatial and contrast resolution of the 3D turbo spin echo images demonstrate an optimal contrast between the high intensity of the cerebrospinal fluid or labyrinthine fluid, and all other structures. The latter are outlined as low-intensity areas, such as cranial nerves, blood vessels, brainstem, cerebellum, and bony surroundings. Maximal intensity processing (MIP) of the 3D volume data acquired by the SPACE sequence produces 3D images of the high-intensity structures of the labyrinth. The 3D MIP of the SPACE sequence enables fast identification of abnormalities at the level of the SCCs. Narrowing or signal loss at the level of the SCC on MRI was defined when there was <50% of normal SCC diameter compared to the normal side or in consensus in case of bilateral pathology (Figure 2).

### Statistics

Pearson chi-square tests were used to compare categorical variables. A logistic regression (ANOVA) was used for correction for age. A *p*-value of 0.05 or less was considered statistically significant.

## Results

Forty-five patients with P51S mutations in the *COCH* gene were included in the study (overall, 51% females and 49% males). In total, 90 ears were investigated. Missing data of caloric testing, audiometry, CT, and MRI results are provided in Tables 1, 2. Age at caloric testing during ENG ranged from 44 to 77 years with a mean of 62 ± 10 years. Mean and median time interval between ENG and CT or MRI was 3 and 25 days, respectively. Two patients had their imaging 3 and 4 years after their first ENG. More than half of patients (54%) were candidates for cochlear implantation.

**Table 1:** Overview of audiometry and caloric testing results with the missing data in all 90 ears.

	***N***	**Normal/abnormal**
Caloric testing	*n* = 52 Missing, *n* = 38	Normal, *n* = 9 (17%) Hypofunction, *n* = 43 (83%)
Audiometry	*n* = 50 Missing, *n* = 40	Non-CI candidates, *n* = 23 (46%) CI candidates, *n* = 27 (54%)

**Table 2:** Correlation between lateral semicircular canal function loss (caloric testing) or pure tone audiometry and imaging abnormalities of the three SCC on CT and MRI.

	**The different SCC**	***n***	**Audiometry (*p*-value)**	**Caloric testing (*p*-value)**
CT *n* = 23 Missing, *n* = 67	Any abnormality	*n* = 21 (91%)	0.001	0.025
	Lateral	*n* = 20 (87%)	0.001	0.025
	Posterior	*n* = 5 (22%)	<0.001	NS
	Superior	*n* = 4 (17%)	0.001	NS
MRI *n* = 38 Missing, *n* = 52	Any abnormality	*n* = 38 (100%)	NS	0.042
	Lateral	*n* = 35 (92%)	NS	NS
	Posterior	*n* = 32 (84%)	NS	NS
	Superior	*n* = 33 (87%)	NS	NS

*NS, not statistically significant difference*.

**Figure 3:**
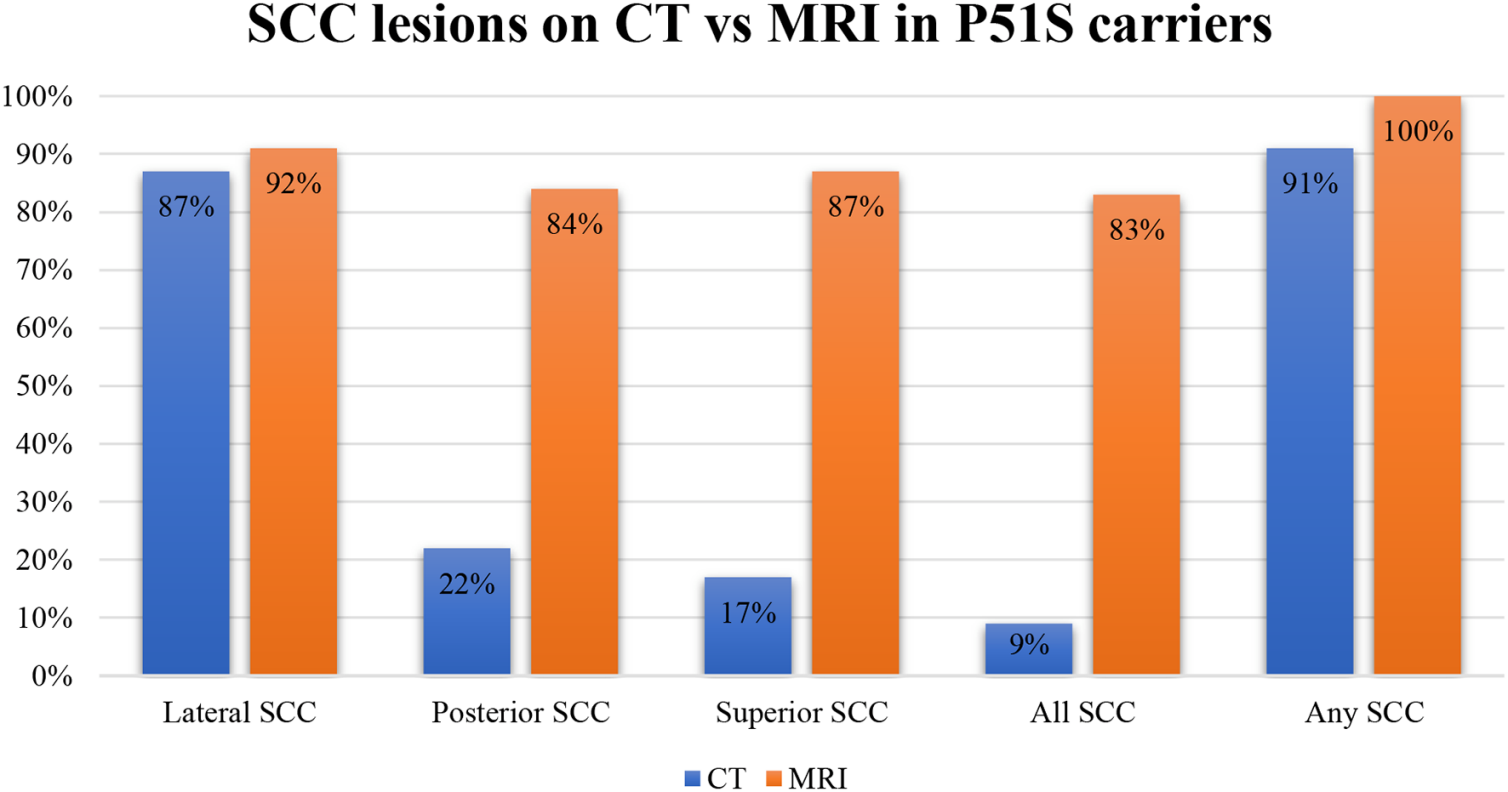
A bar plot illustrating the percentages of the affected semicircular canals on CT and MRI.

In our population, 91% of tested ears had sclerotic lesions and/or narrowing in one or more SCCs on CT scan. All tested ears had SCC narrowing or signal loss in at least one SCC on T2-weighted MRI. Moreover, in 83% of tested ears, all three SCCs were affected on MRI. The number affecting all SCCs per year on CT was 10%. On CT, 22% of tested ears had abnormalities of the posterior SCC, while 17% had abnormalities of the superior SCC and 87% had abnormalities of the lateral SCC. On MRI, 84% had abnormalities in the posterior canal, 87% had abnormalities in the superior canal, and 92% had abnormalities in the lateral canal (Figure 3).

We tested correlations between the ears with severe hearing loss >85 dB HL, therefore CI candidates, with CT and MRI. There was no correlation between audiometry and abnormalities on MRI in one of the SCCs. However, there was a correlation between audiometry and CT imaging abnormalities. This was statistically significant when there was an abnormality in one of the SCCs (*p* = 0.001) as well as in the posterior (*p* < 0.001), superior (*p* = 0.001) and lateral canal (*p* = 0.001) separately (Tables 1, 2).

Moreover, 77% of tested ears showed a bilateral and 11.5% showed a unilateral hypofunction on ENG while 11.5% showed a normal result. CT abnormalities correlated with caloric hypofunction. This statistically significant difference was present if abnormalities were observed in at least one of the SCCs (*p* = 0.025) as well as in ipsilateral lateral SCC function loss (*p* = 0.025). MRI abnormalities in at least one of the SCC correlated with caloric hypofunction (*p* = 0.042), but there was no direct correlation with lateral SCC abnormalities on MRI and ENG (Tables 1, 2). Finally, there was no statistically significant correlation between advanced age and the presence of caloric areflexia or the presence of any abnormalities on temporal bone CT scan or MRI.

## Discussion

During the last decades, many genotype–phenotype observational reports with auditory and vestibular testing have shown limited variability across subjects and helped to characterize DFNA9 (5, 13, 14). Recently, JanssensdeVarebeke et al. described a correlation between hearing loss and radiological abnormalities in patients with DFNA9, specifically with a P15S mutation in the *COCH* gene (9). This study suggested that radiological findings and the degree of hearing loss are both linked to the stage of this hereditary disease (9). However, vestibular function testing was not reported. Therefore, the radiological abnormalities observed in DFNA9 might also reflect the presence of vestibular function loss. Overall, correlations between vestibular function and imaging were not studied in the work by JanssensdeVarebeke et al.

Our analysis demonstrated a statistically significant correlation between CT and MRI abnormalities at the level of the SCC and caloric areflexia and hypofunction on ENG in patients with DFNA9. These imaging abnormalities are therefore of potential interest as biomarkers associated with a vestibular decay in DFNA9 patients. The only other published radiologic abnormality was reported in one patient with DFNA9 mutation presenting with bilateral superior semicircular canal dehiscence (SSCD) on CT imaging (4). While another case of SSCD was identified on histopathology in a DFNA9 patient, probably a circumstantial finding unrelated to DFNA9 (15).

A possible hypothesis for the radiologic abnormalities has been described as protein misfolding and eosinophilic containing deposits (6, 16). Another hypothesis suggests that these findings are the results of early stage fibrosis followed by end-stage ossification secondary to a slowly progressing inflammatory reaction. The study by JanssensdeVarebeke et al. demonstrated that 31% of the lesions on MRI were not detected on CT imaging, suggesting fibrosis (without sclerosis) at these sites (9). It is noteworthy that no enhancement of the SCC can be observed after administration of gadolinium on T1-weighted images. Our analysis also demonstrated sclerotic lesions and/or narrowing in one or more SCCs on CT scan in 91% of patients while all patients had SCC narrowing or signal loss on T2-weighted MRI. Therefore, MRI is more sensitive for detecting these radiologic abnormalities. Still, CT shows more significant correlations with audiometry as well as ENG results. These results might be explained by the pathophysiology of the fibrosis process. Possibly only the patients with more severe cochleovestibular deterioration show abnormalities on CT. Meaning that the process is started with fibrosis (sensitive on MRI) and ends with ossification (also easily seen on CT). Although this might be a possible explanation of the pathophysiology, there might be a contradiction with our correlation results. If fibrosis (which is easily seen on MRI) is the pathophysiology that explains the abnormal caloric responses in patients with DFNA9, we would expect strong correlations between these vestibular testings and MRI and not CT as we found (which easily shows the process of ossification).

**Figure 4:**
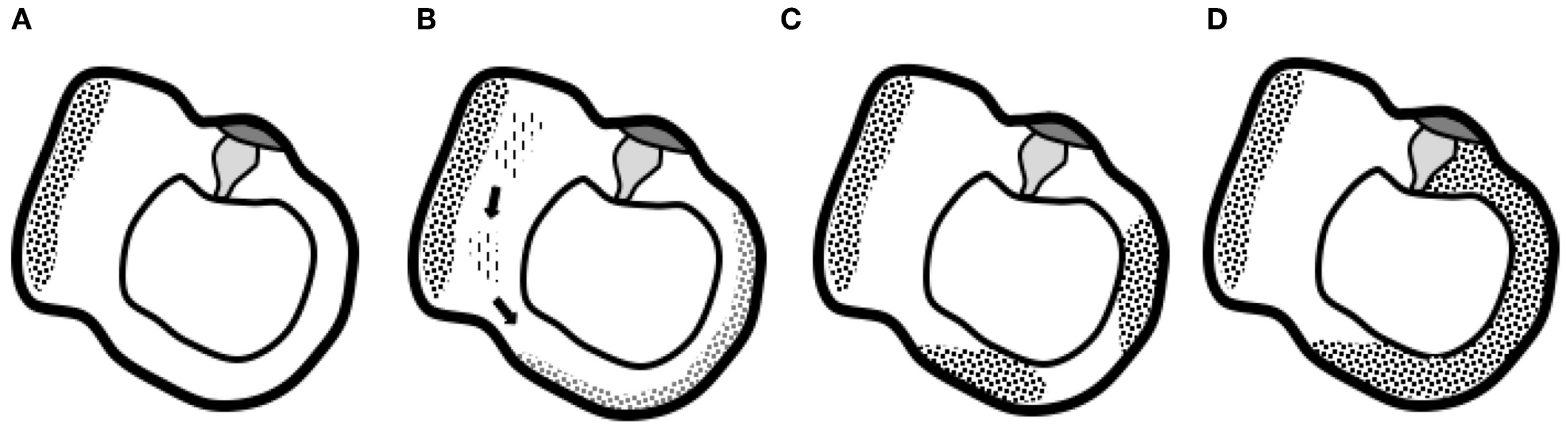
Graphical representation of the suggested hypothesis. **(A)** Normal situation of the utricle and lateral semicircular canal with the utricle's macula and semicircular canal's cupula. **(B)** Progressive degeneration of the membranous labyrinth in the utricle might produce proteins that dislocate and accumulate in the semicircular canal. **(C)** Deposits eventually completely obstruct the semicircular canal and prevent the cupula from detecting angular acceleration of endolymph **(D)**, the end-stage ossification (18).

Quesnel et al. described mechanisms that could explain delayed and progressive sensorineural hearing loss due to fibrous tissue deposition after hearing preservation cochlear implantation (17). They hypothesized that fibrous and bony tissue growth result in an increase in round window impedance and an occlusion of pressure outlets in the scala tympani. Afterwards, the pressure difference drops (pressures in both scalae are high) and a loss of input pressure drive occurs, finally causing sensorineural hearing loss (17). Van Rompaey et al. already suggested a correlation between these described fibrous tissue deposits and vestibular function loss (18, 19). Their study in patients with bilateral severe-to-profound SNHL, eligible for cochlear implantation, but excluding DFNA9 patients, showed abnormalities on T2-weighted MRI correlating to caloric areflexia. Thus, narrowing and/or signal loss in one or more SCCs is a finding not only in patients with DFNA9 but also in patients with vestibular hypofunction/areflexia without known hereditary etiology. The hypothesis suggested by Van Rompaey et al. (18) translates the theory put forward by Quesnel et al. from the cochlea to the vestibular system. More specifically, a progressive accumulation of fibrous tissue deposits toward the SCC membranous ampulla can decrease the impedance of the cupula and the convective current of the endolymph produced by caloric irrigation, which does not stimulate vestibular hair cells to send an afferent signal. Thus, the cupula is obstructed in its movement without the need for vestibular hair cell degeneration. Possibly, degeneration at the level of the utricle results in aggregates of protein that dislocate and accumulate at the cupula, which prevents it from detecting angular acceleration of endolymph (Figure 4) (18). A progressive accumulation of fibrous tissue deposits toward the SCC membranous ampulla can decrease the impedance of the cupula and the convective current of the endolymph produced by caloric irrigation, which does not stimulate vestibular hair cells to send an afferent signal. Alternatively, the impedance in the crista ampullaris may also be increased by remodeling of the extracellular matrix. Anyway, the cupula is obstructed in its movement and the apparent hypofunction is due to a hydrodynamic, mechanical cause, without the need for degeneration within the sensory epithelium.

Limitations of our study are the absence of a control group with unilateral or bilateral caloric areflexia without DFNA9. Another limitation is that caloric testing only evaluates lateral SCC function in its low frequencies, while video Head Impulse Testing (vHIT) can provide data on the function of all SCC in its high frequencies. Rabbitt has described that focal obstruction of the membranous labyrinth would not eliminate higher-frequency responses, which has already been found after superior canal dehiscence plugging. In our study, we described acceleration sensitivity severely affected by complete occlusion of the lumen in the SCC, which would only hold for low-frequency stimuli like calorics. Therefore, a follow-up study using vHIT to test higher-frequency responses would be interesting (20). Moreover, all our patients had a symptomatic P15S *COCH* mutation; thus, future research should investigate correlations between hearing loss, vestibular testing, and imaging in mutations other than P51S. Finally, we did not subgrade our abnormalities of the SCC seen on imaging; we only defined more than 50% of the loss of the canal diameter as abnormal. Future studies using (semi-)automated analysis of the SCC would be helpful to confirm these findings.

To conclude, our retrospective analysis confirms the presence of CT and MRI abnormalities in DFNA9 patients that carry the P51S mutation in the *COCH* gene. A correlation between these radiologic features and vestibular function (tested by means of caloric response) was observed in this population and might also be a biomarker of vestibular decay in patients with P51S *COCH* mutation.

This might be interesting for future research using medical imaging data for algorithms such as Radiomics. Finally, in the future, these results could help diagnose patients in a pre-symptomatic stage as well as patients with normal hearing and bilateral vestibulopathy.

## Data Availability Statement

The datasets generated for this study are available on request to the corresponding author.

## Ethics Statement

The studies involving human participants were reviewed and approved by local ethics committee of the Antwerp University Hospital/University of Antwerp (protocol number 16/42/426). Written informed consent for participation was not required for this study in accordance with the national legislation and the institutional requirements.

## Author Contributions

GM collected the data. SD and VV analyzed the CT and MRI and defined the abnormalities. BI did the analysis of the data. BI and VV drafted the manuscript. SJ and PV revised and corrected the manuscript.

### Conflict of Interest

The authors declare that the research was conducted in the absence of any commercial or financial relationships that could be construed as a potential conflict of interest.
